# Novel nanotech antioxidant cocktail prevents medical diagnostic procedures ionizing radiation effects

**DOI:** 10.1038/s41598-021-84596-w

**Published:** 2021-03-05

**Authors:** Miguel Gorenberg, Abed Agbarya, David Groshar, Ilya Volovik, Ofir Avitan, Igor Sukhotnik

**Affiliations:** 1 Department of Nuclear Medicine, Bnai-Zion Medical Center, Golomb 47, P.O.B 4940, Haifa, Israel; 2 Oncology Unit, Bnai-Zion Medical Center, Haifa, Israel; 3 Department of Nuclear Medicine, Belinson Hospital, Rabin Medical Center, Petah Tikva, Israel; 4 Medical Imaging Department, Bnai-Zion Medical Center, Haifa, Israel; 5 Urology Department, Bnai-Zion Medical Center, Haifa, Israel; 6 Department of Pediatric Surgery, Dana-Dwek Children’s Hospital, Tel-Aviv Sourasky Medical Center, Tel-Aviv, Israel; 7 Technion-Israel Institute of Technology, The Ruth and Bruce Rappaport Faculty of Medicine, Haifa, Israel

**Keywords:** Medical research, Oncology, Risk factors

## Abstract

Ionizing radiation (IR) exposure results in oxidative damage causing cytotoxic and genotoxic effects. Double-strand breaks (DSBs) are considered the most significant DNA lesions induced by ionizing radiation. The present study evaluates the radio protective effect of a novel antioxidant cocktail through quantification of DSB in peripheral blood lymphocytes (PBL) in vivo. The study included 16 consecutive patients who were divided into 2 groups, 6 patients received the novel antioxidant cocktail and 10 control patients. Blood samples were drawn from the patients undergoing bone scan, before the injection of the ^99m^Tc MDP tracer and 2 h after the injection. Quantification of the IR damage was done by Immunofluorescence analysis of the phosphorylated histone, γ-H2AX, used to monitor DSB induction and repair in PBL. The radiation effect of the control group was measured by 2 variables, the average DBSs foci per nucleus and the percent of the DSB bearing cells in PBL. The findings showed a significant increase in the DSBs after isotope injection with an average increment of 0.29 ± 0.13 of foci/nucleus and 17.07% ± 7.68 more DSB bearing cells (*p* < 0.05). The cocktail treated group showed a lower difference average of − 2.79% ± 6.13 DSB bearing cells. A paired *t*-test revealed a significant difference between the groups (*p* < 0.005) confirming the cocktail’s protective effect. The novel anti-oxidant treatment decreases the oxidative stress-induced DNA damage and can be considered as a preventative treatment before radiation exposure.

## Introduction

During the past decades, the growth and development in the field of medical imaging has expanded substantially, mainly due to a rapid technology evolution. Simultaneously, those technologies showed an adaptively rising usage with unavoidable radiation exposure increment. In 2006, the National Council on Radiation Protection and Measurements (NCRP) reported that the increase was mostly due to the higher utilization of computed tomography (CT) scans and nuclear medicine procedures, estimated to be 67 million and 18 million in the US alone, respectively^1^. The mechanism of deleterious ionizing radiation (IR) action is strongly associated with initiation of oxidative stress in irradiated tissues^2^. Another effect of IR that has been proven to be strongly associated with carcinogenesis is inappropriate DNA repair processes of single-strand and double-strand breaks (DSBs) resulting in DNA changes, which in turn can lead to mutations and subsequent cancer^3^. DNA double-strand breaks (DSBs) are considered the most relevant lesion for mutations and carcinogenesis. Unrepaired or miss-repaired DSBs are a great threat to genomic integrity^4–7^. Completeness of the genome might be at risk due to various physical alterations which may disrupt the equilibrium that maintains the cell’s function. Environmental factors, such as radiation, can cause DNA injury and instability hence contribute to imbalance of genetic integrity. Growing evidence suggests that one of the biggest hazards posed by oxidative stress is the generation of DNA damage and, in particular DSBs^8^.

The aim of the present study was to propose an antioxidant based pharmaceutical agent that can be administered before the radiological examination, as an approach to minimize the radiation exposure carcinogenic effects. This antioxidant compound provides both the physicians and patients, the benefits of advanced diagnostic studies with a significant reduced risk of carcinogenesis. The unique nano-encapsulated cocktail contains ten antioxidants and antioxidant-rich plant extracts, including water-soluble and lipid-soluble molecules. Thus, this approach allows achieving a comprehensive and hopefully synergistic impact against radiation damage in vivo in humans.

## Materials and methods

### Participants

The study complies with the Declaration of Helsinki and was performed following approval (BNZ-0070-17) by the ethics committee of the Bnai Zion Medical Center. Written informed consent was obtained from every patient. 16 patients undergoing Technetium-99 m Methylene Diphosphonate (^99m^Tc MDP, Soreq Nuclear Research Center, Israel) bone scans met the inclusion criteria and were consecutively enrolled. Patients were excluded if they received radiotherapy or chemotherapy in the past 6 months, if they underwent imaging using IR in the previous week, or if they consumed nutraceuticals used in the experimental therapy on the same day or before participation.

### Study design and treatment

In this single-center prospective controlled study, the first 10 consecutively recruited participants were assigned to the control group, and the next 6 patients were assigned to the antioxidant group (Table 1). The antioxidant group received an innovative drinkable cocktail of non-toxic radical-scavenging antioxidants, which was developed at the Technion—Israel Institute of Technology, Faculty of Biotechnology and Food Engineering. The components of the nanotech antioxidants cocktail included: Yerba mate, Gingko biloba, N' acetyl cysteine, Epigallocatechin-3-gallate, Quercetin, Curcumin, D-alpha-tocopherol (VE), Ascorbic acid, Vitamin D (VD), and Astaxanthin (AST) (Table 2). The antioxidant cocktail lyophilized dry powder was freshly reconstituted and dissolved in 2000 ml filtered water by the medical personnel and given to the patients. Blood was taken before cocktail ingestion, and 2 h after the ^99m^Tc MDP injection, to compare in vivo post-exposure with pre-exposure foci levels.

**Table 1:** The study patients’ demographic characteristics.

Assigned group	Index	Age [Y]	Gender	Radiation dose [mBq]	Scan indication	Past medical history
Control group	1	50	m	925	Backache	
	2	46	f	925	Arthalgia	
	3	66	f	925	Osteolytic lesions	
	4	19	m	925	Spondylosis	
	5	48	f	925	GIST	
	6	31	m	925	Arthalgia	
	7	44	m	925	Mono-Arthritis	Gout
	8	26	m	925	Osteolytic lesions	
	9	49	m	925	Arthalgia	
	10	69	m	925	Prostate cancer staging	
Antioxidant group	1	47	f	925	Arthalgia	Asthma, TB at childhood
	2	38	f	925	Arthalgia	
	3	50	m	925	Lymphoma	Lymphoma, Schizophrenia
	4	18	f	925	Arthritis	
	5	41	m	925	Arthalgia	
	6	45	f	925	Cervical Mass	

The Control group (n = 10) included three female and seven male participants aged 19–69 years old, having a median of 47 years old. The Antioxidant administered group (n = 6) was comprised of four female and two male subjects with an age range between 18 and 47 years old and a median of 43 years. All participants received the same radiation dose during the scan procedure.

**Table 2:** Composition of antioxidant drink administered to patients undergoing bone scan.

No.	Compound	Type	Quantity (mg)
1	Curcumin	Hydrophobic	80
2	Quercetin	Hydrophobic	150
3	VE	Hydrophobic	750^a^
4	VD	Hydrophobic	1^b^
5	AST	Hydrophobic	40
6	AA^c^	Hydrophilic	2000
7	NAC^d^	Hydrophilic	240
8	EGCG^e^	Hydrophilic	630
9	Yerba mate	Hydrophilic	80
10	Ginkgo biloba	Hydrophilic	320

A ten ingredients cocktail, combining five water insoluble antioxidant compounds, nanotech encapsulated, with five water soluble molecules, was prepared and reconstituted in 2000 ml water as an oral drink given to patients during nuclear medicine diagnostic imaging bone scan procedure.

^a^Equivalent to 830 IU.

^b^Equivalent to 40,000 IU.

^c^Ascorbic acid.

^d^N′ acetyl cysteine.

^e^Epigallocatechin-3-gallate.

**Figure 1:**
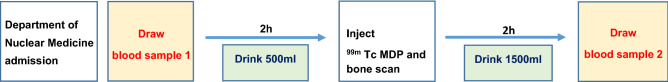
Bone scan patient’s timeline protocol flow chart. Upon admission to the department of Nuclear Medicine the first blood sample (3 ml) was drawn to serve as baseline background values. All bone scan patients received 500 ml (antioxidant rich cocktail or water for control group) to drink within two hours. Later, the patients followed standard bone scan protocol of ^99m^Tc MDP injection and imaging. Then, an addition of 1500 ml antioxidant rich cocktail or water drink for control group, were orally administered. A second blood sample was drawn 2 h after the radioisotope injection.

All individuals received a 500 ml oral drink: water (control group) or cocktail (antioxidant group) 2 h before the injection, and 1500 ml immediately after the injection of the radioisotope agent. A (3 ml) blood sample was drawn before the injection of ^99m^Tc MDP tracer for the bone scan and a second blood sample was obtained 2 h later after scintigraphy imaging (Fig. 1). The quantity of DNA damage was compared between the blood samples obtained before and after the injection of the radioactive tracer, to assess for any significant difference, for both control and antioxidant treated groups.

### Assessment

Immunofluorescence analysis of the phosphorylated histone, γ-H2AX, was performed to monitor DSB induction and repair in vivo in PBL^9–11^. The assay protocol followed the procedure by Löbrich et al.^9^ Briefly, blood lymphocytes were isolated by centrifugation on Ficoll gradient to enable layer separation. The resulted white blood cells were fixed, mounted on coverslips and incubated with monoclonal mouse anti human γ-H2AX antibody. The lymphocytes were washed and incubated with goat-anti mouse Alexa Fluor 488 conjugated providing green fluorescence. After subsequent washing, the cover slips were mounted by Vectastain medium with 4′,6-diamidino-2-phenylindole (DAPI) to visualize blue-fluorescence background nuclei. Imaging was facilitated through a Zeiss fluorescent microscope where each slide was assessed counting 40 cell nuclei. This method allows the estimation of DSBs number and location after irradiation doses that are typically associated with medical imaging^12,13^. It is considered a very reliable and sensitive technique^14^.

Detection and quantification of focal DNA lesions has been achieved using three-dimensional microscopy. The DSBs foci were counted before and after ^99m^Tc MDP medical radioisotope tracer administration. A direct comparison between the control and the antioxidant groups has been made.

### Statistical analysis

The data was processed and analyzed using SPSS version 20 software. A paired *t*-test was used to show the harmful effect of the IR on the control group. The comparison between groups was done by using a one-way ANOVA test. *p*-value below 0.05 was considered a significant result.

## Results

No side effects have been reported by the recruited participants of the current trial.

**Figure 2:**
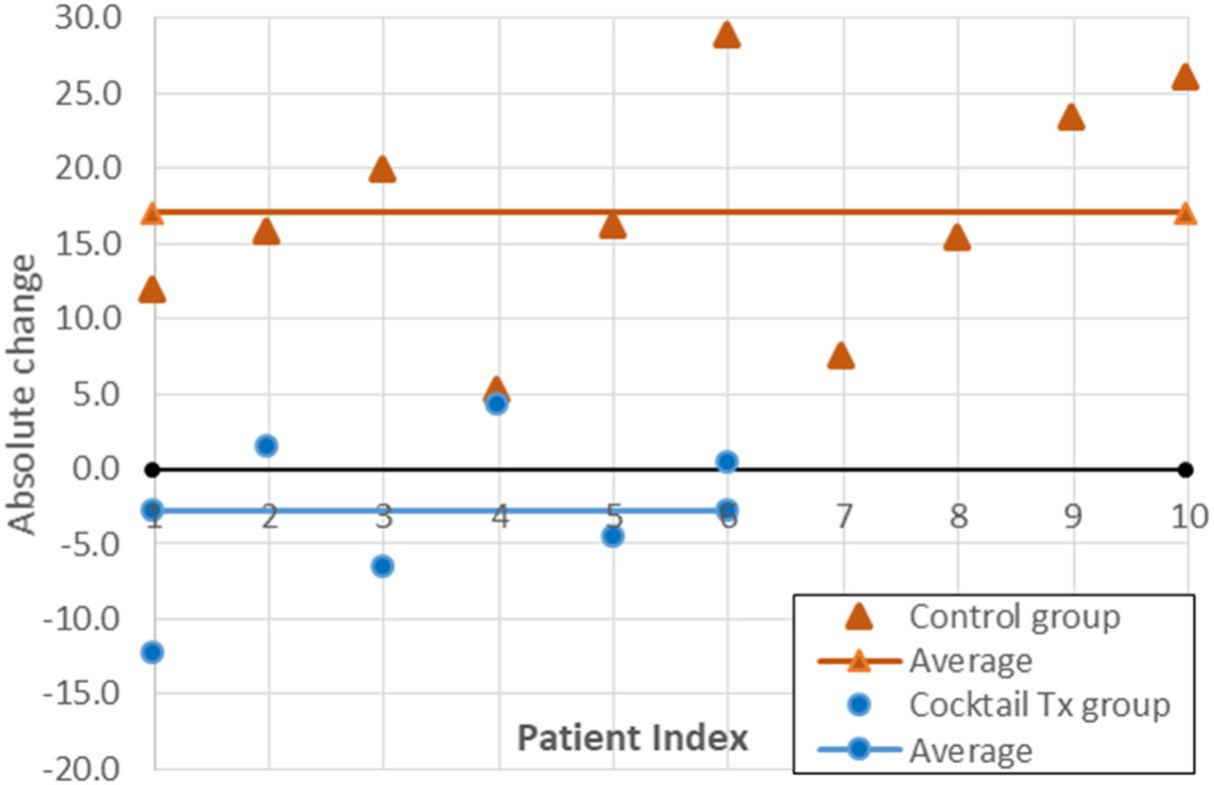
Individuals' distribution of the difference in % DSBs bearing cells in PBL expressed as an absolute change, calculated after 2 h of radiation exposure. The number of DNA DSBs in control and treatment groups was measured by immunofluorescence microscopy of gamma-H2AX. Control group (orange triangles) differences average 17.07 and cocktail treated group (blue circles) − 2.79 were marked as horizontal lines, in their respective colors. The baseline average of % DSBs bearing cells (before radiation exposure) was considered zero for each group and is marked as black line.

To present the impact of radiation and the cocktail’s benefit, the average of DSBs foci per nucleus in each cell was calculated and the percent of the DSBs bearing cells in the PBL was determined. The control group results verified that the radiation effect caused a significant difference in the DSB absolute change after isotope injection. The increase was found both at the foci /nucleus level (0.29 ± 0.13) and the percent of DSBs bearing cells in PBL (17.07% ± 7.68) (*p* < 0.05) (triangles in Fig. 2).

The antioxidant treated group showed a notably low average of − 2.79% ± 6.13 DSBs bearing cells as absolute change after isotope injection compared to pre-radioisotope injection (circles in Fig. 2).

**Figure 3:**
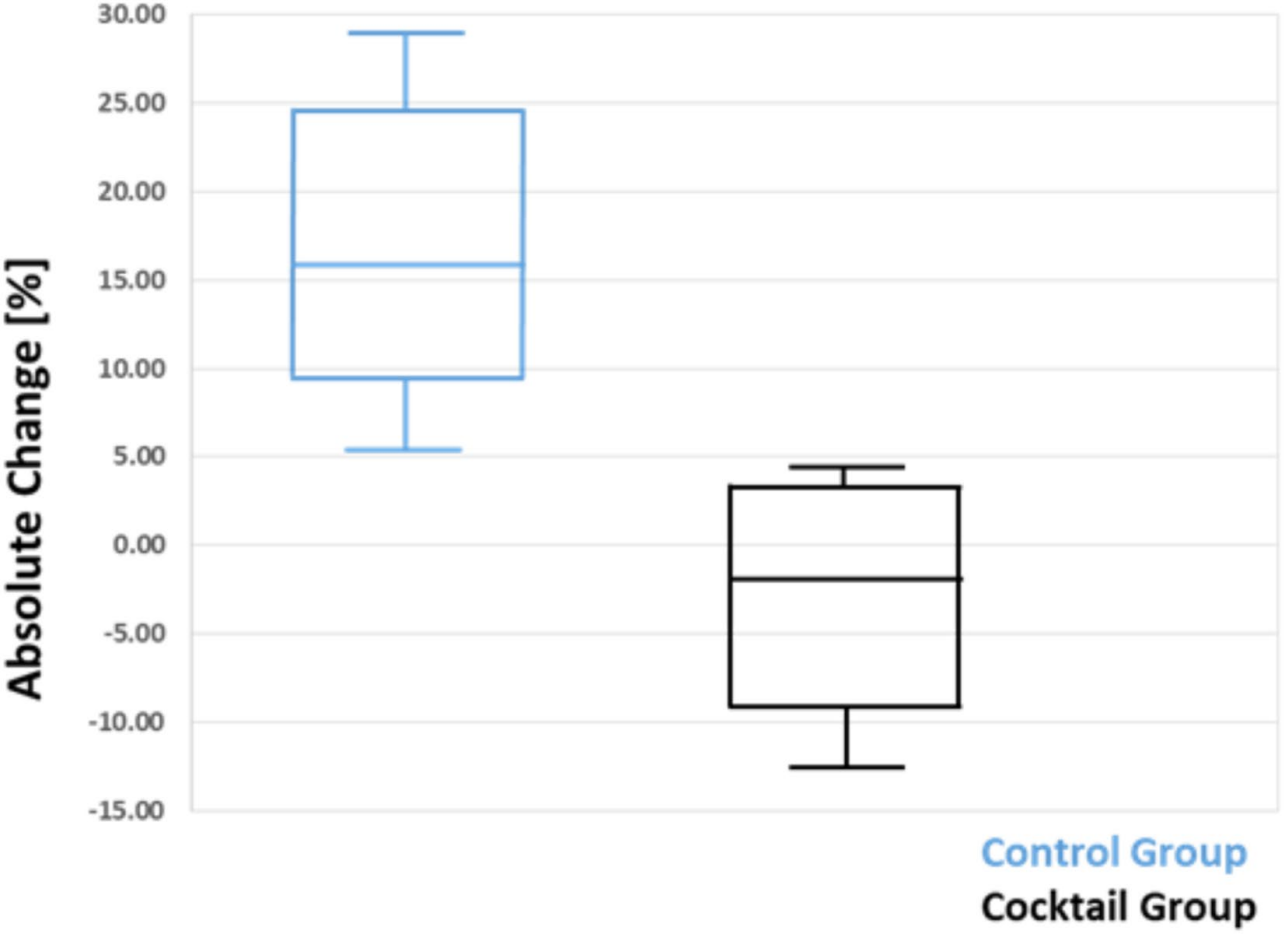
Box Plot of the Absolute Change of % DSBs bearing cells in Control group (blue) versus Cocktail group (black). The median of each group is represented as a horizontal line. Bars indicate the highest and lowest value of each group. There was a statistically significant difference between patients treated with oral antioxidants nanococktail compared with control patients after the radiation study time point (*P* = 0.005).

Comparison between groups using paired *t*-test revealed a significant difference between patients group treated with antioxidant cocktail and untreated patients control group, *p* < 0.005 (Fig. 3).

## Discussion

The underlying biological and molecular mechanisms of radiation-induced damage are complex and still not fully understood, resulting in potentially improper radiation protection. Growing evidence suggests that the biological effect of IR can be mainly related to DNA damages^15^. While acute effects of radiation are related to oxidative stress and reactive oxygen species (ROS) production, radiation-induced damage may be responsible for the long-term biological effects of radiation. The pathway of radiated photon absorption involves interactions with intracellular molecules, particularly water. This reaction produces primary ion radicals that degenerate into highly reactive free hydroxyl radicals. The hydroxyl radical accounts for about two thirds of all DNA damage^16^.

Antioxidants are proposed as biological protection agents against radiation damage in humans^17^. Antioxidants can act at several different stages in an oxidative pathway process; by scavenging initiating radicals, quenching or scavenging singlet oxygen, and breaking the chain of an initiated radical transfer sequence^18^. The multiple antioxidant defense mechanisms available within the cell and extracellularly should be adequate to protect against oxidative damage. However, despite the plethora of protecting mechanisms, it might be insufficient in case of ROS overproduction. This could happen as a consequence of exposure to sources that overwhelm the antioxidant defense, such as high levels of X-ray and gamma ray radiation^18^. Therefore, the extent of tissue damage is the result of the balance between the free radicals generated and the antioxidant protective defense system^19^.

Antioxidant-based radioprotection is well explored and tested in vitro and in vivo for its effectiveness in preventing radiation hazardous damage^20–22^.

Recently, several publications have shown the deleterious effects to health caused by radiation exposure of patients undergoing diagnostic procedures^23^. Interventional radiologists have been found to have cataract incidence up to 5 times higher than unexposed individuals in medical professions of the same age and sex^24^. Furthermore, a causal relationship between chronic exposure to radiation and the development of radiation-induced tumors was suggested^25^. A recent case study documented 31 individual cases of interventionists diagnosed with various brain and neck tumors, showing: 17 professionals affected with glioblastoma multiforme, five with meningiomas, two with astrocytomas^26^.

The main goal of the current study was to evaluate in vivo whether a drinkable nano-cocktail, combining ten non-toxic radical-scavenging antioxidants, administered prior to irradiation may hopefully decrease the formation of DSBs and DNA lesions, which are the main mechanisms of cancer induction after IR. New antioxidant cocktail includes selected vitamins, minerals and herbs with research proven radioprotective effect: Curcumin, Quercetin, Vit E, Vit D, Ascorbic acid, N’- acetyl cysteine, Epigallocatechin-3-gallate, Yerba mate, Ginkgo biloba. All of these compounds are commonly used in clinical practice and all of them have been investigated for their metabolism in the human body. Minimal toxicity effect has been described for every compound. Yerba mate (*Ilex paraguariensis*) contains a variety of polyphenols such as the flavonoids quercetin and rutin, which has been reported to possess a high quantity of caffeoylquinic acids that may be beneficial for other applications instead of its conventional use as a hot beverage^27^. Extracts from Ginkgo biloba leaves include biflavonoids (like Amentoflavone) that have many biological activities, such as antioxidant, anti-inflammatory, anti-bacterial, antiviral, hypoglycemic, anti-tumor and inducing apoptosis^28^.

In a recent study, we investigated the radioprotective effect of similar antioxidant cocktail (vit A, Biotin, vit C, vit D3, vit E, vit B1, vit B2, vit B3, vit B6, folic acid, vit B12, Selenium, N-acethyl cysteine (NAC), Coenzym Q (Ubiquinone), herbal blend that include Ginkgo biloba, *Ilex paraguriensis*, Lycopene, Quercetin, Spirulina) on germ cell apoptosis and spermatogenesis in rats subjected to whole body radiation^29^. We have demonstrated a significant decrease in plasma Malondialdehyde (MDA) levels following antioxidant cocktail administration in rats, suggesting less excessive ROS production and inhibition of oxidative stress. No toxicity has been observed in experimental animals in this study. Similarly, no side effects have been reported by all recruited participants in the current trial.

The approach of this study was to form potato protein nanoparticles (NPs) that entrap the five water-insoluble compounds separately (quercetin, curcumin, VE, VD, and AST). The NPs protect these antioxidants from being destroyed during preparation, lyophilization, storage, digestion, mask undesired flavors and enhance their bioavailability for maximal protective efficacy. The protective effect of this antioxidant cocktail was examined by evaluating the formation of ionization induced DSBs DNA lesions in patients undergoing bone scans. The positive effects of antioxidant mixtures in preventing DNA damage and DSBs production have never been previously described.

This study has demonstrated a significantly protective effect of new antioxidant cocktail in reducing DSB’s rate. While control (non-treated with antioxidant agents) patient group demonstrated a significant increase in the DSBs levels after isotope injection (when compared to DSBs levels before isotope injection), the antioxidant treated group showed a notably low average of DSBs bearing cells compared to initial levels. Statistical analysis (using paired *t*-test) revealed a significant difference between patients treated with antioxidant cocktail and non-treated patients.

IR-induced DNA damage initiates the signaling the transduction pathway (known as the DNA damage response) that results in activating multiple cellular signaling molecules involved in determining the cell fate, cell cycle arrest, apoptosis, autophagy and DNA repair. The most lethal forms of DNA damage after IR exposure are DNA double-strand breaks^30^. Our data suggest that the use of antioxidants may diminish IR-induced tissue damage both by direct inhibition of oxidative stress and by standard repair processes of damaged DNA.

This study has several limitations. First, ^99m^Tc MDP bone scan was chosen as the source of patients’ exposure to IR, whereas there are other medical imaging modalities causing radiation exposure such as CT scan. Second, our study included a limited number of patients and was designed as a single center prospective study.

## Conclusion

The present data suggest the use of a simple and easy to treat antioxidant cocktail via oral intake, which can be used as a protective measure to lessen the iatrogenic radiation effects. Large prospective study is needed to further evaluate the protective effect in different settings to establish a future therapy and standard protocols.

## Data Availability

All the data for this study will be made available upon reasonable request to the corresponding author.
